# Role of tendon-derived stem cells in tendon and ligament repair: focus on tissue engineer

**DOI:** 10.3389/fbioe.2024.1357696

**Published:** 2024-08-08

**Authors:** Wei He, Chao Jiang, Ping Zhou, Xujun Hu, XiaoPeng Gu, SongOu Zhang

**Affiliations:** ^1^ Shaoxing People’s Hospital, Shaoxing, Zhejiang, China; ^2^ Department of Clinical Medicine, Health Science Center, Ningbo University, Ningbo, Zhejiang, China; ^3^ Department of Orthopedics, Zhoushan Guhechuan Hospital, Zhoushan, Zhejiang, China

**Keywords:** tendon-derived stem cell, tendon injury, tissue engineer, nanotechnology, seed cell

## Abstract

This review offered a comprehensive analysis of tendon and ligament injuries, emphasizing the crucial role of tendon-derived stem cells (TDSCs) in tissue engineering as a potential solution for these challenging medical conditions. Tendon and ligament injuries, prevalent among athletes, the elderly, and laborers, often result in long-term disability and reduced quality of life due to the poor intrinsic healing capacity of these avascular structures. The formation of biomechanically inferior scar tissue and a high rate of reinjury underscore the need for innovative approaches to enhance and guide the regenerative process. This review delved into the complexities of tendon and ligament structure and function, types of injuries and their impacts, and the limitations of the natural repair process. It particularly focused on the role of TDSCs within the context of tissue engineering. TDSCs, with their ability to differentiate into tenocytes, are explored in various applications, including biocompatible scaffolds for cell tracking, co-culture systems to optimize tendon-bone healing, and graft healing techniques. The review also addressed the challenges of immunoreactivity post-transplantation, the importance of pre-treating TDSCs, and the potential of hydrogels and decellularized matrices in supporting tendon regeneration. It concluded by highlighting the essential roles of mechanical and molecular stimuli in TDSC differentiation and the current challenges in the field, paving the way for future research directions.

## 1 Introduction

Tendon and ligament injuries are common among athletes, the elderly, and individuals engaged in physical labor, comprising a significant portion of musculoskeletal disorders (Leong et al., 2020). These injuries not only cause pain and dysfunction but can also lead to long-term disability and a substantial decrease in quality of life. The complex structure and biomechanical properties of tendons and ligaments make them particularly challenging to heal, with current treatments often failing to fully restore the original strength and functionality (Cottrell et al., 2016). This highlights the clinical importance of developing more effective repair strategies.

The intrinsic healing capacity of tendons and ligaments is notably poor due to their avascular nature, which restricts the influx of cells and nutrients to the injury site (Andarawis-Puri et al., 2015). Consequently, the repair process is slow and often leads to the formation of scar tissue, lacking the original tissue’s biomechanical properties. As a result, there is a high rate of reinjury and an extended recovery period. These challenges necessitate innovative approaches that can enhance and direct the regenerative process, ensuring the restoration of the tendon or ligament to its native state (Nichols et al., 2019).

Tissue engineering has emerged as a promising field offering potential therapeutic strategies for the repair and regeneration of damaged tendons and ligaments (Lim et al., 2019). By incorporating principles from cell biology, material science, and engineering, tissue engineering aims to develop biological substitutes that can restore, maintain, or enhance tissue function (Ruiz-Alonso et al., 2021). This multidisciplinary approach often involves the utilization of stem cells (Tevlin et al., 2016), biocompatible scaffolds (Stace et al., 2018), and bioactive molecules (Atienza-Roca et al., 2018) to create an environment conducive to healing. With the capability to differentiate into tenocytes, the cells responsible for maintaining tendon structure and function, TDSCs are at the forefront of research as a cellular source for tissue-engineered constructs (Zhang et al., 2023). This literature review will explore the role of TDSCs within the context of tissue engineering and investigate how this synergy might pave the way for effective tendon and ligament repair.

## 2 Basic science of tendon and TDSCs

### 2.1 Structure and function of tendons and ligaments

**FIGURE 1:**
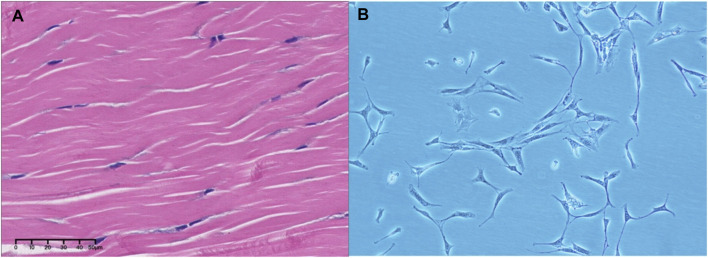
Morphology and structure of tendons and TDSCs. **(A)** Morphological picture of tendon tissue; **(B)** Morphological picture of TDSCs. **(A,B)** are pictures obtained by the author in previous research.

**FIGURE 2:**
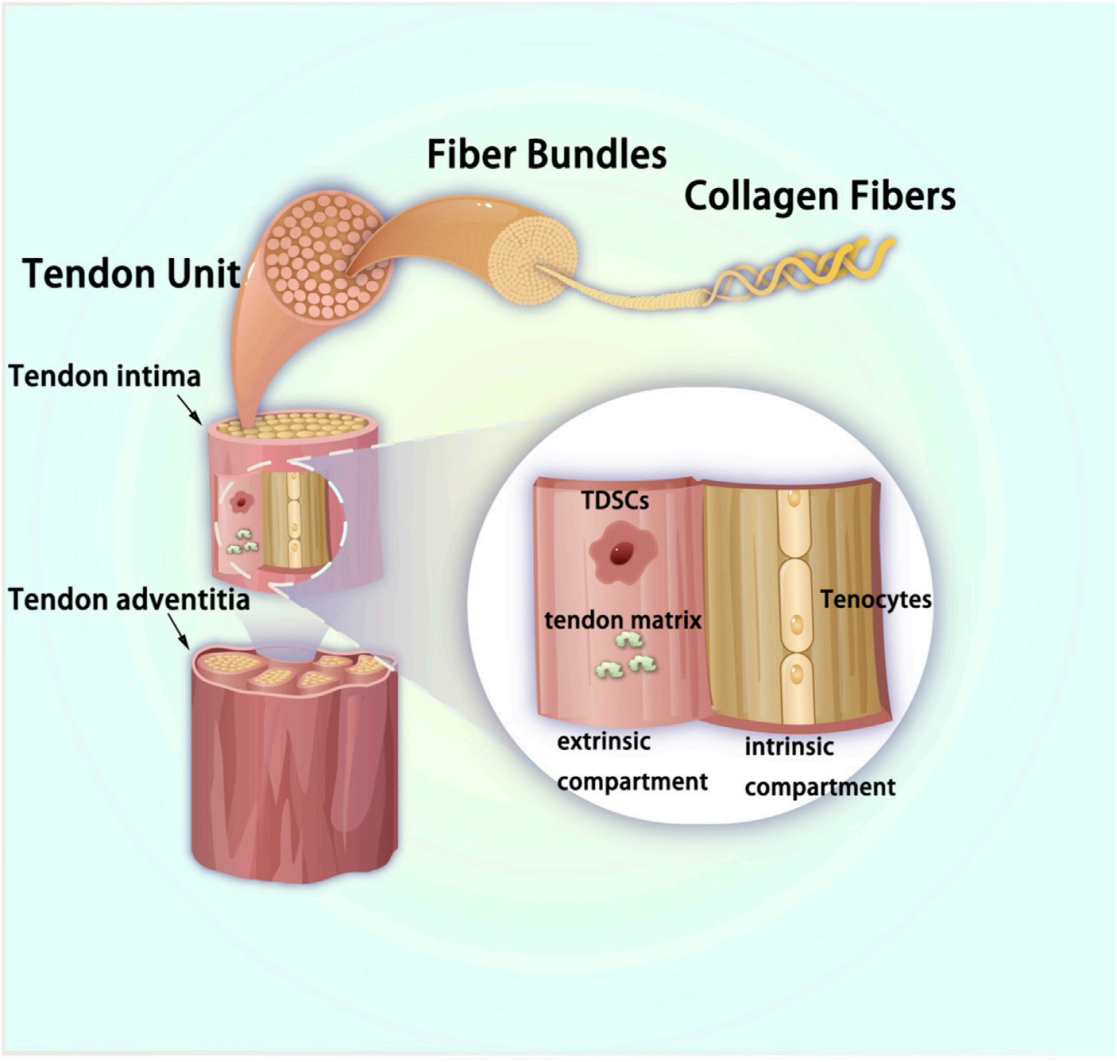
Pattern picture of tendon tissue.

Tendons and ligaments are vital components of the musculoskeletal system, serving as crucial connectors within the body. Tendons attach muscle to bone, enabling movement by transmitting forces generated by muscle contractions to the skeleton. Ligaments connect bones to each other, offering joint stability and guiding motion. Both structures consist of dense fibrous connective tissue, primarily composed of collagen fibers, which provide tensile strength and elasticity (Thorpe and Screen, 2016). The main structural component of tendons is type I collagen (Col I), a protein arranged in a highly ordered manner that forms the tendon’s primary load-bearing structure. Collagen molecules themselves have an elongated triple helix structure, and these molecules are further packed tightly together to form microfibers. Microfibers aggregate into thicker fiber bundles, and these fiber bundles are then aggregated to form large fiber bundles visible in tendons. These thicker fiber bundles can be called tendon clusters. Tendon clusters are composed of tendon inner chambers and tendon outer chambers. The inner compartment is composed of tenocytes and multiscale assembled collagen clusters, and the outer compartment is synovium-like tissue that connects blood vessels and the nervous system. Each level of structure increases the overall strength and elasticity of the tendon (Franchi et al., 2007; Thorpe and Screen, 2016). As show in Figures 1A, 2. The main cell types in tendons are tenocytes and tendon-specific stem cells which we evaluate in this review as TDSCs. These cells are distributed between collagen fibers and are responsible for synthesizing and breaking down collagen, thus participating in the maintenance, repair and regeneration of tendons. Tenocytes are flat and aligned parallel to the direction of collagen fibers, which helps them transmit mechanical and chemical signals efficiently throughout the tendon. In addition to collagen fibers and tenocytes, tendons also contain a certain amount of matrix and interstitium, which are filled between cells and fibers (Chen et al., 2022). The matrix is primarily composed of proteoglycans and glycoproteins, which are highly absorbent and help maintain tendon lubrication and serve as a medium for nutrient transport (Subramanian and Schilling, 2015). The presence of interstitium is critical to the overall health and function of the tendon, providing a supportive environment that helps maintain the tendon’s structural stability and transmit force. The microstructure of tendons shows a hierarchical tissue structure, from single collagen molecules to microfibers, to fiber bundles, and finally to the entire tendon. This hierarchical structure enables tendons to have extremely high mechanical properties, which can not only withstand high-intensity tensile forces, but also have a certain degree of elasticity, ensuring effective force transmission between muscles and bones (Zabrzyński et al., 2018). This distinctive composition and alignment enable tendons and ligaments to endure substantial mechanical stress during daily activities and athletic pursuits (Zitnay and Weiss, 2018).

### 2.2 Definition and characteristics of TDSCs

From a developmental perspective, tendon tissue originates from the mesoderm in the embryo. Some mesoderm cells separate and concentrate in the ectoderm. These cells differentiate into bones, cartilage, tendons, and other connective tissues. Cells characterized by Sox9 positive expression in tendon tissue are considered to be the source of TDSCs. One of the defining characteristics of TDSCs lies in their capacity for self-renewal, which denotes their ability to replicate while retaining an undifferentiated state—an essential aspect for continual tissue maintenance. These cells exhibit multipotency, suggesting their potential to differentiate into various cell types, primarily tenocytes, but also potentially including adipocytes, chondrocytes, and osteocytes under specific conditions (Bi et al., 2007; Rui et al., 2010; Liu Q. et al., 2018). The morphological characteristics of TDSC mainly show spindle or fibroblast morphology (Figure 1B). The initial growth rate is slow, and it generally reaches 40%–50% confluence about 10 days after separation (Zhang Z. et al., 2021). Identification of TDSCs involves specific surface markers, such as Scleraxis (Scx), a critical transcription factor in tendon development, and Mohawk (Mkx), another tendon-specific transcription factor. Additional markers include tenomodulin (Tnmd), a type II transmembrane glycoprotein, and CD44, a cell surface glycoprotein involved in cell-cell and cell-matrix interactions. These markers are used to isolate and identify TDSCs from tendon tissues. Moreover, markers such as CD146, CD105, CD90, Oct4, SSEA-4, and Nucleostemin are also employed for identifying TDSCs (Yang et al., 2016; Lee et al., 2018; Jo et al., 2019; Li et al., 2021; Wei and Lu, 2021). At the cellular marker level, the molecular markers of TDSCs closely resemble those of bone marrow mesenchymal stem cells (BM-MSCs). However, TDSCs specifically express tendon markers such as Scx, Mkx, Tnmd, and Col I (Zhang et al., 2018). Similar to BM-MSCs, TDSCs possess differentiation potential toward osteoblasts, chondrocytes, tendons, and adipocytes. Notably, TDSCs exhibit enhanced chondrogenic and tenogenic differentiation capabilities (Jiang et al., 2023). Following tendon injury, the altered local microenvironment may induce aberrant differentiation of TDSCs, leading to impaired tendon repair (Zhang and Wang, 2014). TDSCs play a pivotal role in maintaining tendon homeostasis by contributing to the continuous turnover of the extracellular matrix within tendons, thereby ensuring structural integrity and functionality. Furthermore, they exhibit responsiveness to mechanical stimuli, a crucial aspect given the primary function of tendons in force transmission (Wang HN. et al., 2020). Changes in mechanical loading can influence TDSC behavior, impacting both their proliferation and differentiation.

Upon tendon injury, TDSCs become activated, migrating to the site of damage. Subsequently, they proliferate and differentiate to replace lost or damaged cells, playing a fundamental role in the initial stages of tendon repair. However, their response may occasionally lead to aberrant healing, characterized by scar tissue formation, highlighting the necessity for precise modulation of TDSC activity to optimize tendon repair (Lui and Chan, 2011; Huang et al., 2021).

TDSCs exhibit substantial promise in the realms of regenerative medicine and tissue engineering. These cells can be harvested, cultured, and potentially subjected to genetic modification to augment their regenerative potential. They are explored in various therapeutic approaches, including stem cell injections (Yin et al., 2018; Chen et al., 2021), tissue-engineered constructs (Zhang and Cheng, 2013), and gene therapy (Lai et al., 2022), to improve tendon repair and function. TDSCs play an integral role in tendon biology, contributing significantly to both the maintenance and repair of tendon tissues. Their capacity for self-renewal and differentiation, combined with their responsiveness to mechanical stimuli, positions them as pivotal targets in strategies designed to improve tendon regeneration and repair (Harvey et al., 2019). Understanding the detailed characteristics and behaviors of TDSCs is imperative for advancing therapies related to tendons and ameliorating outcomes in tendon injuries and disorders.

### 2.3 Methods for isolation and culture of TDSCs

The isolation of TDSCs entails enzymatic digestion of tendon tissue to release the cells, followed by employing cell sorting techniques like flow cytometry to isolate stem cell markers. The digestive juice currently used to digest tendon tissue is mainly type I collagenase (Randelli et al., 2016a) or type II collagenase (Brown et al., 2014). Upon isolation, TDSCs are cultured in specialized media that sustains their growth and stemness. These conditions often incorporate a three-dimensional scaffold mirroring the native tendon environment, thereby fostering natural behavior and differentiation potential in the cells. However, reported culture conditions exhibit variance across studies. While the most commonly used medium is low-glucose. Dulbecco’s modified Eagle medium (DMEM) medium (Chen et al., 2012; Qiu et al., 2019; Ni et al., 2021), others include high-glucose DMEM medium (Brown et al., 2014; Rajpar and Barrett, 2020), α-MEM medium (Feng et al., 2020), or DMEM/F12 medium (Song et al., 2017). Moreover, experiments commonly supplement with 10%–20% fetal bovine serum (Randelli et al., 2016b; Yin et al., 2019; Lai et al., 2022; Ni et al., 2023). In addition to culture media and serum, researchers often add certain small molecule substances to the culture system to promote cell growth. The most common ones are ascorbic acid (Popov et al., 2015a) and L-glutamine (Ni et al., 2012). A few researchers also add Growth factor fibroblast growth factor-2 (FGF2) (Di Meglio et al., 2020) or connective tissue growth factor (CTGF) (Lui et al., 2016). However, there is currently no absolutely unified standard for the training system, which may be related to the habits of researchers and the specific purpose of the experiment.

Progress in culture techniques, exemplified by dynamic bioreactors applying mechanical stimulation, has notably amplified both the proliferation and tenogenic differentiation of TDSCs, yielding a more physiologically relevant model for investigating tendon biology and regenerative therapies. Table 1 illustrates 10 representative cell isolation and culture conditions. Recent research has indicated that 3D culture systems favor the growth and differentiation of TDSCs, establishing them as a pivotal entry point for tissue engineering (Hsieh et al., 2018; Yin et al., 2020).

**TABLE 1:** Ten representative cell isolation and culture conditions.

Cell source	Digestive enzymes	Digestion time	Medium	FBS (%)	Reference
Human	3 mg/mL type I collagenase and 4 mg dispase II	1.5 h	α-MEM	20	Randelli et al. (2016a)
Human	4 mg/mL type I collagenase	2 h	LG-DMEM	10	Qiu et al. (2019)
Rat	3 mg/mL type I collagenase	2 h	HG-DMEM	10	Han et al. (2019)
Rat	3 mg/mL type I collagenase	2 h	LG-DMEM	20	Li et al. (2019)
Rat	3 mg/mL type I collagenase	2.5 h	HG-DMEM	10	Wang et al. (2020b)
Mice	1% type II collagenase	0.75 h	HG-DMEM	10	Brown et al. (2014)
Mice	1.5 mg/mL II collagenase and 2 mg dispase II	0.5 h	α-MEM	10	Feng et al. (2020)
Rabbit	3 mg/mL type I collagenase	Not given	LG-DMEM	10	Ni et al. (2021)
Rabbit	0.25% collagenase	Overnight	LG-DMEM	10	Shen et al. (2012)
Horse	Collagenase (details not given)	Overnight	HG-DMEM	10	Rajpar and Barrett (2020)

FBS, represent fetal bovine serum; LG, represent low glucose; HG, represent high glucose.

## 3 Application of TDSCs in tendon tissue engineering

TDSCs represent a distinct subset of stem cells located within tendons, characterized by their unique ability to differentiate into tenocytes, the fundamental cells responsible for maintaining tendon structure and function. The domain of tendon tissue engineering endeavors to harness the regenerative potential of these cells to mend or substitute damaged tendon tissue, posing a considerable challenge owing to the restricted healing capabilities of tendons. Tables 2–4 provides an overview of ongoing research concerning TDSCs in tendon tissue engineering.

**TABLE 2:** Application of TDSCs in tissue engineering seed cell optimization.

Cell source	Stimulus method	Assessment methods	Main evaluation index	Main finding	Reference
Rat	TSG-6 knockdown	Biomechanical Testing	Failure load	The ultimate stress was greater in the TDSCs group (4.91 ± 1.41 N/mm^2^) as compared with the Control group (2.99 ± 1.04 N/mm^2^) (*p* < 0.05). The TSG-6 silenced group (3.36 ± 0.96N/mm^2^) showed no benefit over the control group	Cheng et al. (2014)
Rat	FGF-2 overexpression	Biomechanical Testing	Failure load	The stiffness of the Achilles tendon in Control group, Vector group, and FGF-2 group at 4 weeks postoperative were 3.87 ± 0.63 N/m, 6.72 ± 1.72 N/m, and 16.21 ± 1.97 N/m	Guo et al. (2020)
Rat	Trim54 overexpression	Biomechanical Testing and Morphological analysis	failure load and histological scores	Overexpressed TRIM54 in these injured rats and observed a rescue effect in morphology and Biomechanical Testing. However, the author did not give specific values	Chen et al. (2024)
Rat	Chip overexpression	cytological experiments	proliferation and differentiation ability	Overexpression of Chip can significantly increase the proliferation and tenogenic differentiation ability of TDSCs	Han et al. (2017a)
Rat	TNF-α, TGF-β	cytological experiments	differentiation ability	The combined use of 5 ng/mL TGF-β1 and 0.0025 ng/mL TNF-α can significantly increase the differentiation ability of TDSCs	Han et al. (2017b)
Rat	different matrix stiffness	cytological experiments	proliferation and differentiation ability	The higher the matrix stiffness, the stronger the proliferation and differentiation ability of TDSCs in culture	Liu et al. (2018b)
Rat	Different concentrations of IL-10	cytological experiments	migration and differentiation ability	10 ng/mL concentration of IL-10 significantly inhibited the migration and differentiation ability of TDSCs	Deng et al. (2018)
Rat	Cell sheet with BMP and TGF-β gene intervention	Radiology, biomechanics	failure load and bone formation	These results indicate that the Ad-BMP-2/TGF-β1-transfected TDSC sheet promotes biomechanical strength and reduces inflammatory infiltration	Wei et al. (2023)
Rat	TGIF1 knockdown	Morphological analysis	Cartilage-related staining	Knockdown of Tgif can promote chondrogenic differentiation of TDSCs	Chen et al. (2015)
Human	CITED2 knockdown	Cytology and Molecular Biology Experiments	β-gal staining	Downregulation of CITED2 contributes to TGFβ-mediated senescence in TDSCs	Hu et al. (2017)
Rat	AuNC-siRNA	Cytological and biomechanical experiments	β-gal staining and failure load	Gold nanoparticles can improve inflammation-induced aging by blocking the IKKβ/NF-κB pathway. It can promote an increase in failure load of 10% in aged tendons	Wang et al. (2022)
Rat	Spironolactone	Cytological and morphological experiments	β-gal staining and heterotopic ossification	Spironolactone (1 μM and 10 μM) can improve TDSC aging and tendon heterotopic ossification. Its mechanism may be through the NF-κB/MAPK Pathway	Xu et al. (2021a)
Rat	Low oxygen environment	Cytology and Molecular Biology Experiments	alizarin red staining and Osteogenic differentiation related indicators	Hypoxic environment (3% O_2_) can inhibit the osteogenic differentiation of TDSCs	Li et al. (2016)
Rat	Co-culture with BM-MSC	Cytology and Molecular Biology Experiments	Cell proliferation ability and Osteogenic differentiation related indicators	1:1 Co-culture can increase the proliferation capacity of TDSCs compared with single culture	Li et al. (2016)
Rat	Co-culture with BM-MSC	Cell and animal experiments	Cell proliferation and tendon differentiation ability	In the group with a cell ratio of 1:1, TDSCs had stronger proliferation and tenogenic differentiation abilities	Wu et al. (2016)
Human	Co-culture with ADSC	Cytology and Molecular Biology Experiments	tendon differentiation ability	On the seventh day of co-culture of tendon stem cells and adipose-derived mesenchymal stem cells, type I collagen deposition increased significantly	Costa-Almeida et al. (2018)
Mice	TGF-β, FGF-4 treatment	Cytology and Molecular Biology Experiments	tendon differentiation ability	Similar responses as TPCs to specific treatments suggest MSCs have tenogenic potential	Brown et al. (2015)
Mice	Tnmd Knockout	Cytology and Molecular Biology Experiments	β-gal staining and tendon differentiation ability	Tnmd knockout causes TDSC to be susceptible to aging but does not affect their differentiation ability	Alberton et al. (2015)

**TABLE 3:** Summary of TDSC research in the field of mechanical stress.

Cell source	Mechanical stimuli type	Stimulation frequency	Elongation	Stimulation time	Research focus	Main finding	Reference
Rat	UCMT	1 Hz	4%	0.5 h	Mechanism of cystic fibrosis transmembrane conductance regulator regulating tendon stem cell differentiation	Cystic fibrosis transmembrane conductance regulator plays an important role in tenogenic differentiation and tendon regeneration by inhibiting the b-catinin/pERK1/2 signaling pathway	Liu et al. (2017)
Rat	UCMT	0.5 Hz	4% or 8%	4 h	Does mechanical stimulation affect TDSC osteogenic differentiation	Repetitive tensile loading increased the expression of BMP-2 and addition of BMP-2 enhanced osteogenic differentiation of TDSCs	Rui et al. (2011)
Rat	UCMT	0.5 Hz	2%	4 h	Mechanism of heterotopic ossification after overuse of tendon tissue	UMT induced osteogenic differentiation of rTDSCs via the Wnt5a-RhoA pathway	Shi et al. (2012)
Rabbit	UCMT	0.5 Hz	4% or 8%	12 h	Exploring the mechanobiological responses of TDSCs	Low mechanical stretching (4%) may be beneficial to tendons by enabling differentiation of TSCs into tenocytes to maintain tendon homeostasis. However, large mechanical loading (8%) may be detrimental, as it directs differentiation of TSCs into non-tenocytes in tendons	Zhang and Wang (2010)
Rat	UCMT	0.3 Hz, 0.5 Hz, and 1.0 Hz	2%, 4%, and 8%	3 h/day for 7 days	Exploring the effects of different stretch intensities on the proliferation and differentiation of TDSCs	Stretching had a significant effect on type I collagen, tenascin-C, tenomodulin, and scleraxis of TDSC, especially at 0.5 Hz frequency with 4% amplitude	Xu et al. (2015)
Rat	UCMT	1 Hz	8%	48, 60, or 72 h	Exploring the causes of heterotopic ossification after tendon overuse	Osteogenic differentiation of TDSCs via the Wnt5a/Wnt5b/JNK signaling pathway	Liu et al. (2015)
Rat	UCMT	0.5 Hz	8%	12 h	Exploring the effect of PRP on TDSCs under strong tension	PRCR promotes tenocyte differentiation while inhibiting adipocyte, chondrocyte, and osteocyte lineages, which are believed to hinder tendon healing	Chen et al. (2012)
Rat	UCMT	0.2 Hz	10%	48 h	To investigate the effect of mechanical stress on the co-culture system of BMSCs and TDSCs	Mechanical stimulation enhances the regenerative potential of BMSCs and TCs in tendon-bone healing by promoting the proliferation and differentiation of local precursor cells	Song et al. (2017)
Rat	UCMT	0.5 Hz	4 or 8%	2 h	To explore the effects and mechanisms of excessive mechanical stimulation on TDSCs *in vitro*	Mechanical loading activates mTOR signaling in TDSCs	Nie et al. (2021)
Mice	treadmill running	3 h/day, 4 h/day, and 5 h/day	/	5 days/week	Exploring the responses of TDSCs and mature tenocytes in tendons to mechanical stress	mTOR maintains tendon homeostasis by promoting TDSC differentiation into tenocytes. In contrast, improper tension causes tendinopathy by inducing non-tenocyte differentiation in TSCs	Zhang et al. (2020)
Human	UCMT	1 Hz	1%, 5% or 8%	1 h/day for 3 days	Comparison of the effects of different mechanical stimulation protocols on TDSCs	8% mechanical loading had a positive effect on matrix proteins, integrins and matrix metalloproteinases, and activation of integrin downstream kinases p38 and ERK1/2 in TDSCs	Popov et al. (2015b)
Mice	3D UCMT	0.25 Hz	6%	8 h	Establishing a protocol to simulate the stress effects of TDSCs during tendon development	This protocol,6% strain, 0.25 Hz, 8 h followed by 16 h rest for 6 days, could mimic cell differentiation in the tendon	Chen et al. (2020)
Mice	UCMT and 3D UCMT	0.25 Hz	6%	8 h	Comparison of the effects of uniaxial and biaxial mechanical stretching on TDSCs	3D cell niches are essential for tendon tissue development	Wang et al. (2018)

UCMT, represent uniaxial cyclic mechanical tension.

**TABLE 4:** Application of TDSC in scaffold-based tendon regeneration.

Cell source	Scaffold type	Favorable factors in scaffold	Disease models	Main finding	Reference
Rat	cell sheet	TDSC and ECM	ACL rupture	The TDSC sheet improved early graft healing after ACL reconstruction in the rat model	Lui et al. (2014)
Rat	Decellularized Cell Sheet	ECM	ACL rupture	dTDSC sheets alleviate the quality control and safety concerns of cell transplantation and can be used as a cell-free alternative for the promotion of graft healing in ACLR.	Yao et al. (2023)
Rat	different Culture matrix	nanotopographic cues and substrate stiffness	tendinopathy	The differentiation of TDSCs is affected by the mechanical stiffness and nanotopography of the culture substrate, which has implications for tendon regeneration and healing	Kim et al. (2018)
Rabbit	engineered tendon matrix	TDSC and ECM	Tendon regeneration	ETM can effectively expand TDSCs *in vitro* and improve tendon repair *in vivo*	Zhang et al. (2011)
Rat	Decellularized tendon matrix	TDSC and ECM	Achilles tendon rupture	Tendon-derived decellularized matrices combined with tendon stem/progenitor cells provide a promising strategy for functional tendon tissue regeneration	Song et al. (2018)
*Macaca mulatta*	Decellularized tendon matrix	ECM	Tendon regeneration	T-gel retains the nanofibrous structure and bioactive factors of native tendon ECM, making it a potential hydrogel for tendon regeneration	Ning et al. (2021)
Human	Decellularized matrix (tendon, bone and dermis)	ECM	Tendon regeneration	Implantation of this cell-scaffold construct led to a more mature structure (histology score: 4.08 ± 0.61 vs 8.51 ± 1.66), larger collagen fibrils (52.2 ± 1.6 nm vs 47.5 ± 2.8 nm) and stronger mechanical properties [stiffness: 21.68 ± 7.1 Nm m (-1) vs13.2 ± 5.9 Nm m (-1)] of repaired tendons compared to the control group	Yin et al. (2013)
Rat	Decellularized Small Intestinal Submucosa	ECM	Achilles tendon rupture	Biologically prepared SIS scaffolds synergistically promote tendon regeneration with TDSCs, while achieving anti-adhesion through M2 macrophage polarization	Mao et al. (2022)
Rat	Young Decellularized tendon matrix	ECM	Tendon regeneration	The impaired capacity of aged TDSCs can be rejuvenated by exposure to young DECM	Jiang et al. (2018)
Rat	Synthetic Materials	Bioactive agent (KGN and MBGs)	rotator cuff injury	Bioactive agent-loaded hydrogels add value to biomaterials used in chronic tendon-bone junction injuries	Huang et al. (2022)
Human	Synthetic Materials	3D nanofiber	Achilles tendon rupture	the RADA-based hydrogels exert a rejuvenating effect by recapitulating *in vitro* specific features of the natural microenvironment of human TSPCs, which strongly indicates their potential to direct cell behaviour and overcome the challenge of cell aging and degeneration in tendon repair	Yin et al. (2020)
Human	Synthetic Materials	3D microenvironment	Tendon regeneration	Thermosensitive BC hydrogel holds great potential as an injectable cell delivery carrier of TSPCs for tendon tissue engineering	Yin et al. (2018)
Human	Synthetic Materials	aligned focal contact points	Tendon regeneration	These results showed a novel strategy for directing stem cell behavior without the use of exogenous growth factors or pre-aligned COL I fibers, and propose that anisotropic nanocomposite hydrogels hold great potential for tendon tissue engineering applications	Xu et al. (2021b)
Human	Synthetic Materials	3D RADA peptide	Patellar Tendon rupture	The combination of TSPC and nanofiber hydrogel provide an optimistic alternative method to accelerate functional tendon repair with reduced heterotopic ossification	Zhang et al. (2023)
Rat	Synthetic Materials	Chitosan/β-Glycerophosphate/Collagen	Achilles tendon rupture	The improved healing was indicated by the improvement in histological and immunohistochemistry outcomes and the increase in the biomechanical properties of the regenerated tissue at both 4 and 6 weeks post-injury	Yang et al. (2017)
Rat	Synthetic Materials	chitosan	Tendon regeneration	TSPC-seeded chitosan scaffolds offer a feasible approach for tendon repair	Chen et al. (2018)
Rat	Synthetic Materials	3D nanofiber	Tendon regeneration	Electrospun bundled nanofiber yarns (NFYs) replicate native tendon tissue structure, highlighting their potential as biomimetic scaffolds for tendon regeneration	Yang et al. (2022)
Rabbit	Synthetic Materials	aligned tissue morphology	Achilles tendon rupture	Engineered scaffolds facilitate TDSC proliferation and migration, promote tenogenesis, and enhance mechanical properties, indicating their value in tendon tissue engineering	Ning et al. (2022)
Rat	Synthetic Materials	3D aligned microenvironment	Achilles tendon rupture	3D-aligned TSPCs in a biomimetic topology are promising for functional tendon regeneration	Li et al. (2023)
Rat	Synthetic Materials	Silver nanoparticles	Tendon regeneration	Silver nanoparticles (75–150 μg mL^−1^) have a certain degree of toxicity to tendon stem cells. NAC can reduce the toxicity	Cheung et al. (2016)
Human	Synthetic Materials	3D aligned electrospun nanofiber threads	Tendon regeneration	Key scaffold features mimicking native tissue are crucial for developing engineered tendon substitutes	Laranjeira et al. (2017)
Rat	Synthetic Materials	Silk fibroin	Tendon regeneration	The 10 μm SF film group had the highest percentage of oriented cells and the most significant changes in cell morphology, as well as the highest expression of COL1A1, TNC, TNMD, and SCX.	Lu et al. (2020a)
Rat	Synthetic Materials	Silk fibroin	Tendon regeneration	SF films with a bionic microstructure may serve as a scaffold, provide biophysical cues to alter the cellular adherence arrangement and cell morphology, and enhance the tenogenic gene and protein expression in TSPCs. FAK activation plays a key role during this biological response process	Lu et al. (2020b)
Rabbit	silk-collagen	silk scaffold	rotator cuff injury	Allogeneic TSPC-seeded knitted silk-collagen sponge scaffolds show clinical potential for tendon tissue engineering	Shen et al. (2012)
Human	Synthetic Materials	aligned nanofibers	Tendon regeneration	An aligned electrospun nanofiber structure creates an instructive microenvironment for hTSPC differentiation, useful for engineered tendon development	Yin et al. (2010)
Rat	Synthetic Materials	hBM-MSC secretome with keratin electrospun scaffolds	rotator cuff injury	Human mesenchymal stem cell-conditioned medium (hMSCs-CM) increases hTCs viability and density *in vitro*, and the cells integrated into keratin scaffolds show significant benefits	Sevivas et al. (2018)
Mice	Synthetic Materials	Bioinspired bimodal micro-nanofibrous	Achilles tendon rupture	Micro-nanofibrous scaffolds improve the structural and mechanical properties of regenerated Achilles tendons, presenting significant potential for improving tendon tissue engineering outcomes	Yin et al. (2022)
Rat	Synthetic Materials	bioactive electrospun nanofiber membranes	Tendon regeneration	Bioactive electrospun nanofiber membranes are suitable as biomimetic scaffolds in tendon-bone tissue engineering, enhancing tendon-bone healing	Lin et al. (2019)
Rat	Synthetic Materials	3D printed scaffolds	rotator cuff injury	An *in situ* tissue engineering approach could improve rotator cuff repair outcomes	Tarafder et al. (2019)

### 3.1 Tissue engineering seed cell optimization

Pre-treatment of TDSCs involves subjecting these cells to specific growth factors or environmental cues before transplantation. This process aims to augment the cells’ inherent healing capabilities, rendering them more effective once introduced into the injury site. Furthermore, this approach can be customized to promote specific cellular behaviors, such as increased proliferation or directed differentiation, which are indispensable for facilitating effective tendon repair (Wang et al., 2023).

In the research on TDSCs treatment of tendon-related diseases, the study of molecular mechanisms has always been a hot topic. A clear molecular mechanism can provide a reference for pretreatment of seed cells for tissue engineering. Cheng et al. (2014) intervened the expression of TNF alpha-stimulated gene/protein 6 (TSG-6) in TDSC and found in the experiment that TDSCs can promote the repair of rotator cuff injury. Therefore, TSG-6 plays an important role in TDSCs repairing rotator cuff injuries. In a human TDSC study, TDSCs from fetal Achilles tendons were isolated and cultured to overexpress FGF-2. The researchers found that after overexpression of FGF-2, the expression of tendon-related factors Scx and type III collagen increased. Transplanting FGF-2 overexpressing TDSC into the Achilles tendon notch in rats can promote Achilles tendon healing (Guo et al., 2020). In the process of tendinopathy, inflammation and programmed death are pathological processes that inhibit tendon self-repair. Tripartite Motif Containing 54 (TRIM54) inhibits inflammation and apoptosis in rat TDSCs by stabilizing YOD1 deubiquitinase (YOD1) (Chen et al., 2024).

Promoting tendon directional differentiation of TDSCs is an important research topic in this field. The study found that STIP1 homology and U-box containing protein 1 (STUB1) (Han W. et al., 2017), transforming growth factor-beta 1 (TGF-β1) (Han P. et al., 2017), and focal adhesion kinase- Extracellular signal-regulated kinase 1/2 (FAK-ERK1/2) signaling pathways (Liu C. et al., 2018) are factors that promote the differentiation of TDSC into tendon, while tumor necrosis factor α (TNF-α) (Han P. et al., 2017), interleukin 10 (IL -10) (Deng et al., 2018) and the janus kinase/signal transducer and activator of transcription 3 (JAK/Stat3) signaling pathways (Deng et al., 2018) are inhibitors of this process. In tendon diseases, in addition to the repair of tendon tissue, the repair of the tendon-bone connection is a more important issue. Because in most tendon diseases, such as rotator cuff injuries, cruciate ligament reconstruction, etc., it is necessary to restore the connection and normal physiological gradient between the tendon and bone. TDSCs are often used to study the role of tendon-bone junction repair. The normal tendon-bone insertion gradient is a bone-mineralized fibrocartilage-nonmineralized fibrocartilage-tendon structure. Researchers study the gradient of tendon-bone insertion point repair by TDSCs from different angles and mechanisms. Studies have found that bone morphogenetic protein 2 (BMP-2) and TGF-β1 can promote osteogenic and tenogenic differentiation of mouse TDSC *in vivo* and *in vitro* (Wei et al., 2023). As an upstream signal of TGF-β1, transforming growth interacting factor 1 (TGIF1) plays an inhibitory role in the chondrogenic differentiation process of TDSCs. Manipulating the expression of TGIF1 can promote the repair of tendon-bone junctions (Chen et al., 2015). The aging of stem cells is manifested by the decrease in differentiation and proliferation capabilities, apoptosis or fibrosis, resulting in a decrease in the tendon’s ability to self-repair. Targeting the aging mechanism of TDSCs can provide important targets for the treatment of tendon-related diseases. A study on human-derived TDSCs found that the expression of CBP/p300-interacting transactivator with Glu/Asp-rich C-terminal domain, 2 (CITED2) decreased in aging tendon, and TGFβ2 regulated the aging of TDSC through CITED2 (Hu et al., 2017). Inflammation can induce premature senescence of TDSCs, and the NF-κB signaling pathway plays an important regulatory role in this process (Xu K. et al., 2021; Wang et al., 2022). During tendon repair, excessive osteogenic differentiation can cause complications of heterotopic ossification. In rat TDSCs, ERK1/2 is involved in the osteogenic differentiation of TDSC (Li et al., 2016).

**FIGURE 3:**
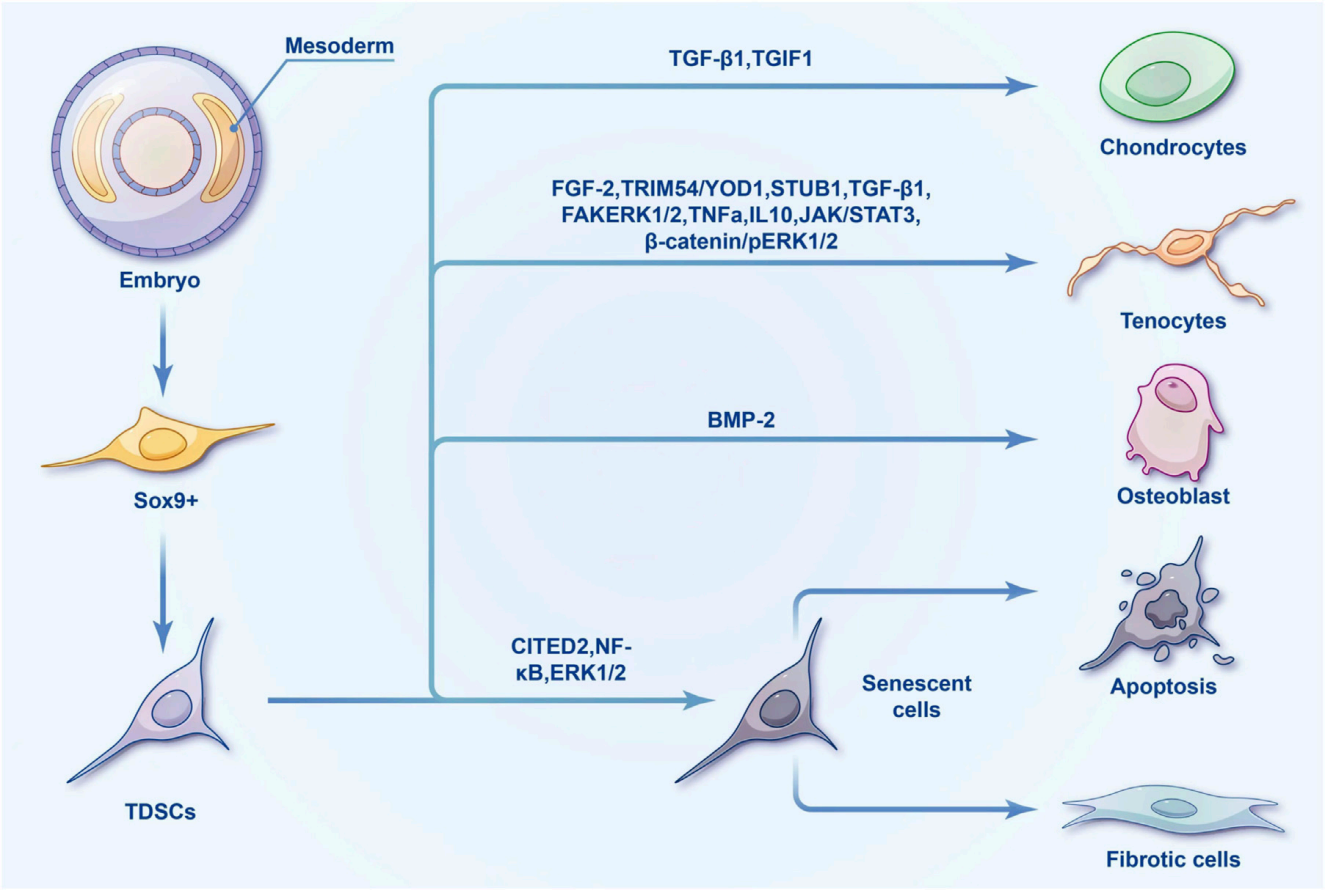
Differentiation tree of TDSCs.

Co-culture systems, involving the simultaneous cultivation of diverse cell types like BM-MSCs and TDSCs, play a pivotal role in enhancing tendon-bone healing in tissue engineering. The study by Liu et al. (2019) demonstrated that optimal co-culture ratios, particularly a 1:1 ratio of BM-MSCs and TDSCs, significantly boost cell proliferation, differentiation, and tenogenic activities. These interactions are further influenced by factors such as Tenascin C, capable of impacting cellular behaviors like osteogenesis and chondrogenesis. Additionally, molecular pathways involving Rho-associated kinase (ROCK), insulin-like growth factor 1 receptor (IGF-1R), and methyl ethyl ketone (MEK) are implicated in these processes, highlighting a complex regulatory network. Another study suggests that a balanced co-culture of BM-MSCs and TDSCs not only enhances proliferation and collagen production but also elevates tenogenic marker gene expression and collagen matrix production, particularly in equal proportion co-cultures (Wu et al., 2016). This approach has been proven to effectively promote tendon healing in a rat model, underscoring its potential as an enhanced cell source for tendon tissue engineering (Figure 3).

Moreover, interactions between human adipose-derived stem cells and tendon cells have shown temporal regulation of tenogenesis, emphasizing the significance of extracellular matrix (ECM) synthesis and remodeling in co-cultured systems. These interactions lead to controlled cell proliferation and elongation, along with an improved ratio of collagen type I to III, crucial for generating high-quality tendon tissue. Co-culture systems in tendon tissue engineering are indispensable for optimizing tendon-bone healing (Costa-Almeida et al., 2018). The synergy between different cell types in co-cultures, coupled with the regulation of specific molecular pathways and ECM remodeling, plays a vital role in enhancing tendon repair and regeneration.

### 3.2 Advances in graft healing techniques

The application of TDSC sheets has significantly advanced graft healing techniques in tendon repair, particularly in anterior cruciate ligament (ACL) reconstruction. These sheets, often combined with growth factors, serve as a direct source of reparative cells and bioactive molecules at the injury site, thereby fostering improved graft integration and healing outcomes. In a rat model, TDSC sheets were employed to encase ACL grafts. Treated with connective tissue growth factor and ascorbic acid, the sheets augmented graft healing by elevating tunnel bone mineral density and bone volume, enhancing osteointegration, and preserving greater intra-articular graft integrity. These findings were supported by the presence of GFP-positive cells, signifying the successful integration of the TDSC sheets into the healing process (Lui et al., 2014). Zhang Y. et al. (2021) implemented a stepwise culture strategy for TDSCs, resulting in a significant upregulation of tendon-related genes and proteins. By integrating this culture system with 3D printing technology, researchers embedded chemically empowered TSPCs within a biocompatible hydrogel to engineer tendon grafts, displaying an enhanced capacity to promote functional tendon repair and regeneration both *in vivo* and *in situ*. Using genetically modified TDSCs to improve cell sheet properties. Wei et al. (2023) found that BMP-2 can promote osteogenic differentiation of TDSC, while TGF-β1 can promote tenogenic differentiation of TDSC. Simultaneous transfection of BMP-2 and TGF-β1 into TDSCs can significantly improve the maturation of tendon-bone junctions. If the Cell sheet is made with allogeneic TDSC, it may cause rejection (Dang et al., 2021). Therefore, decellularization and leaving the extracellular matrix will greatly reduce the possibility of rejection (Inci et al., 2020). Yao et al. (2023) investigated the use of decellularized TDSC (dTDSC) sheets, which retained the bioactive factors of the natural extracellular matrix and fostered graft healing after ACLR. These sheets exhibited heightened bone formation, improved graft osteointegration, and enhanced midsubstance graft integrity. This method also influenced macrophage polarization and MMP/TIMP expression, contributing to better healing outcomes. These advancements underscore the potential of TDSC sheets in enhancing graft healing in tendon repair, particularly in ACL reconstruction. Through leveraging the unique properties of TDSCs and innovative techniques such as 3D printing and decellularization, these approaches offer promising strategies to improve graft integration, facilitate tissue regeneration, and ultimately enhance the outcomes of tendon repair surgeries.

### 3.3 Mechanical stimuli in TDSC differentiation

Mechanical and molecular stimuli play pivotal roles in influencing the behavior and differentiation of TDSCs. Mechanical stimuli, such as stretch or compression, mimic the natural mechanical environment of tendons, while molecular stimuli encompass signaling molecules that can guide cell behavior. Understanding the impact of these stimuli on TSPC differentiation is crucial for devising effective strategies for tendon repair. Several research endeavors elucidate these mechanisms. In their study, Liu et al. (2017) delved into the influence of mechanical stretching on TDSC differentiation, revealing the crucial role of the cystic fibrosis transmembrane conductance regulator (CFTR) in tenogenic differentiation. Their findings indicated irregularities, diminished mechanical properties, and reduced matrix formation in tendon tissues of CFTR-dysfunctional mice. Furthermore, RNA sequencing analysis unveiled abnormal activation of the Wnt/β-catenin signaling pathway in TDSCs from these mice. Notably, inhibiting the pERK1/2 signaling pathway fostered tenogenic differentiation and tendon regeneration, suggesting that CFTR regulates tendon differentiation through the β-catenin/pERK1/2 pathway.

Zhang and Wang (2010) investigated the impact of mechanical stretching on TDSC behavior, observing that low mechanical stretching facilitated TDSC differentiation into tenocytes, potentially maintaining tendon homeostasis. However, excessive stretching led to differentiation into non-tenocyte lineages, contributing to tendinopathy features such as lipid accumulation and tissue calcification. Additionally, Chen et al. (2012) explored the effect of autologous platelet-rich clot releasate (PRCR) on TDSCs subjected to mechanical stretching, finding that PRCR increased TDSC numbers and the production of collagen types I and III and TGF-β1, favoring tenocyte differentiation while suppressing adipocyte, chondrocyte, and osteocyte lineages.

Researchers have also discovered that mechanical stress impacts the tendon-bone healing process. Continuous passive motion (CPM) therapy has been shown to enhance healing by promoting fibrocartilage formation, increasing load-bearing capacity, and up-regulating key genes at the tendon-bone interface. Moreover, mechanical stretch has been found to improve cell proliferation and matrix synthesis in a co-culture of BM-MSCs and TCs, underscoring the regenerative potential of local precursor cells in tendon-bone healing (Song et al., 2017). The interplay between mechanical and molecular stimuli in TDSC differentiation and tendon healing offers valuable insights for future tendon injury management strategies.

For TDSCs, the matrix morphology and surface morphology in the cell culture environment have an important impact on the differentiation of TDSCs. Culturing normal TDSCs on a stiff matrix can promote TDSCs maturation and tendon differentiation (Kim et al., 2018). Preserving the stemness of TDSCs *in vitro* culture is a very important issue (Zhang and Wang, 2013; Chen et al., 2016; Yu et al., 2017). Compared with ordinary plastic culture plates, culturing TDSC on the surface of decellularized tendons can preserve the stemness of TDSC (Zhang et al., 2011). The use of a specially designed parallel microgrooved polydimethylsiloxane (PDMS) membrane to culture TDSCs can guide the elongation of TDSCs. Under such culture conditions, the ability of TDSCs to differentiate into adipogenesis, chondrogenesis, and osteogenesis is significantly inhibited. The ability of tendon differentiation is enhanced (Shi et al., 2017). Therefore, the application of hard, regular, and parallel culture matrix morphology in vitro culture systems may be meaningful for subsequent stem cell treatments.

### 3.4 Scaffold-based tendon regeneration

Scaffold-based regeneration involves the use of engineered structures specifically designed to facilitate cell attachment, growth, and differentiation. Collagen fiber scaffold used in research on tendon-related diseases (Fan et al., 2008; Sun et al., 2014; Tabesh et al., 2022). Ouyang’s team used homemade collagen fiber scaffolds (Chen et al., 2008) as carriers for TDSCs. *In vivo* experiments observed that the combination of collagen fiber scaffolds and TDSCs resulted in significant collagen production and reduced immune rejection (Shen et al., 2012). Compared with randomly arranged collagen fiber scaffolds, parallel-arranged collagen fiber scaffolds can achieve better mechanical properties as carriers of TDSCs (Zheng et al., 2017). Decellularized matrices represent scaffolds derived from natural tissues, wherein cellular components are removed to minimize immunogenicity while retaining the structural and functional cues of the extracellular matrix. Within tendon tissue engineering, these matrices serve as a foundational support guiding TDSC differentiation and promoting tissue regeneration, thus playing a pivotal role in the development of tissue-engineered tendon constructs. Investigative studies have delved into their influence on TDSCs and tendon repair. Collagen matrices extracted from various tissues (tendon, bone, and dermis) facilitated TSPC adhesion and proliferation. Notably, tendon-derived matrix encouraged a tendinous phenotype and suppressed osteogenesis in hTDSCs (Yin et al., 2013). Moreover, engineered tendon matrix (ETM) from decellularized tendon tissues stimulated TSPC proliferation and preserved stemness *in vitro*, subsequently enhancing the formation of tendon-like tissue *in vivo*, underscoring the potential of ETM in tendon healing (Zhang et al., 2011). When combined with TSPCs, decellularized extracellular matrix from porcine tendons demonstrated superior results in promoting tendon regeneration compared to mesenchymal stromal cells (Song et al., 2018). Additionally, decellularized tendon hydrogel from *Macaca mulatta* supported mTDSC proliferation, migration, and tenogenic differentiation, with the native ECM components of T-gel augmenting these behaviors (Ning et al., 2021). The application of decellularized matrices presents promising prospects in tendon tissue engineering, effectively steering TSPC behavior and fostering tendon repair.

Hydrogels utilized in tendon repair constitute water-swollen, cross-linked polymeric networks simulating the extracellular matrix, thus establishing an environment conducive to cell growth and tissue development. Numerous studies have investigated the potential of hydrogel systems combined with TSPCs to enhance tendon healing and regeneration. In a particular study, a sequential culture strategy using small molecules was developed to potentiate TSPCs for enhanced therapeutic applications. Through high-throughput screening, specific small molecules were identified, leading to heightened TSPC proliferation, initiation of tenogenesis, and subsequent maturation. When these chemically empowered TSPCs were embedded within a biocompatible hydrogel using 3D printing technology, they demonstrated improved functional tendon repair and regeneration *in vivo* (Zhang Y. et al., 2021). Another investigation assessed the use of chitosan/β-glycerophosphate/collagen (C/GP/Co) hydrogel in combination with TDSCs for Achilles tendon healing in a rat model. The research illustrated that local application of TDSCs with C/GP/Co hydrogel significantly improved tendon healing compared to control groups, as evidenced by histological, immunohistochemical, and biomechanical assessments (Yang et al., 2017).

Furthermore, Ying et al. (2020) explored the rejuvenation of aged/degenerative human TDSCs using a self-assembling nanofiber matrix composed of RADA peptide hydrogel. This nanocomposite hydrogel supported the survival, proliferation, and rejuvenation of TDSCs, potentially overcoming challenges associated with cell aging and degeneration in tendon repair. Thermosensitive hydrogels, including a butane diisocyanate (BDI)-collagen hydrogel and methylcellulose/polyvinyl alcohol/polyvinylpyrrolidone-based hydrogel, were examined as injectable cell delivery carriers for TDSCs. These hydrogels exhibited biocompatibility, supported TDSC behavior, and induced differentiation toward tenogenic and osteogenic lineages, making them valuable candidates for tendon tissue engineering (Yin et al., 2018). A magnetically-responsive nanocomposite hydrogel composed of collagen type I and aligned iron oxide nanoparticles showed potential for promoting the alignment and tenogenesis of TDSCs, addressing the issue of uniform cell arrangement in tendon tissue engineering (Xu Y. et al., 2021). Mao et al. (2022) utilized TDSCs combined with small intestinal submucosa (SIS) scaffolds to improve Achilles tendon repair. *In vitro*, SIS facilitated TDSC adhesion and tenogenic differentiation. *In vivo*, TDSCs-SIS scaffolds augmented tendon regeneration and reduced adhesion formation through M2 macrophage polarization. An injectable thermosensitive hydrogel containing kartogenin-loaded bioactive glass nanoparticles displayed promise in addressing rotator cuff injuries. It induced chondrogenesis and osteogenesis in TDSCs, promoting fibrocartilage and bone layer regeneration in a rabbit model of chronic cuff tears (Huang et al., 2022). Zhang et al. (2023) employed a RADA peptide hydrogel with human TSPCs for patellar tendon repair. Results demonstrated improved function recovery, enhanced matrix organization, reduced complications, and decreased heterotopic ossification, suggesting a promising approach for clinical tendon repair. The utilization of hydrogel-based approaches in conjunction with TSPCs presents compelling prospects for enhancing tendon repair and regeneration. Collectively, these studies underscored the potential of hydrogel systems to furnish a conducive microenvironment for TSPCs, foster tenogenesis, and ameliorate functional outcomes in tendon tissue engineering and clinical applications.

Allogeneic TSPCs, exhibiting typical stem cell characteristics, were employed in a study focusing on shoulder repair. These cells were seeded onto knitted silk-collagen sponge scaffolds, demonstrating clonogenicity, high proliferation, multidifferentiation potential, non-immunogenicity, and immunosuppressive properties. In a rabbit model, these TSPCs facilitated tendon regeneration by differentiating into tenocytes, diminishing immunological rejection, and augmenting collagen deposition and biomechanical properties (Chen et al., 2012). Silk-Based Materials in Tendon Engineering: Silk fibroin (SF) films, both with bionic microstructures and smooth surfaces, have exhibited promise in tendon tissue engineering. The 10 μm bionic SF film showcased mechanical properties comparable to native tendons. When TSPCs were seeded on these films, they underwent significant changes in cell morphology, prompted the upregulation of tenogenic genes (COL1A1, TNC, TNMD, SCX), and facilitated TSPC adherence and differentiation (Lu et al., 2020a). Furthermore, SF films with bionic microstructures activated focal adhesion kinase (FAK), playing a pivotal role in enhancing TSPC differentiation. In brief, silk-based materials, particularly bionic SF films, present potential for tendon repair by guiding TSPC morphology and promoting tenogenic differentiation through FAK activation (Lu et al., 2020b). These materials, especially SF films with bionic microstructures, demonstrate substantial promise in tendon tissue engineering. They not only guide TSPC morphology but also enhance tenogenic differentiation through FAK activation, making them customizable biomaterials for tendon repair applications.

The domain of TDSCs and tissue engineering is evolving towards a more comprehensive approach integrating advanced biomaterials, cellular therapies, and an understanding of the biomechanical environment. This multifaceted strategy holds significant promise for surmounting traditional challenges in tendon repair and regeneration, offering new pathways for effective treatments of tendon injuries and disorders.

## 4 Clinical study for tendon with stem cells

Although there are currently no clinical studies specifically on tendon stem cells, several clinical studies on stem cells in tendon-related diseases have been initiated. Some of these studies have already reached conclusions. The stem cells used in these clinical studies are primarily derived from bone marrow and adipose tissue. Stem cell therapy has shown good safety in the treatment of tendon diseases. In the treatment of partial tears in the supraspinatus tendon, although stem cell injections did not show significant effects compared to placebo, all participants only reported transient pain at the injection site with no persistent adverse events (Chun et al., 2022). Additionally, the use of adipose-derived stem cells in treating lateral epicondylosis demonstrated no significant adverse effects, confirming their safety (Lee et al., 2015). Preoperative bone channelling combined with stem cell treatment in rotator cuff repair did not result in any adverse events or significant differences in healing rates compared to sham procedures (Lapner et al., 2021). Stem cell therapy has shown potential efficacy in the repair of tendon ruptures. Adipose-derived stem cells significantly improved structural outcomes in rotator cuff repair, although there were no clinical differences during a 28-month follow-up period; MRI data indicated a significantly lower retear rate in the treatment group compared to the control group (Kim et al., 2017). Moreover, bone marrow-derived stem cells used in the acute repair of achilles tendons showed no reruptures and allowed earlier resumption of walking and sporting activities (Stein et al., 2015). In another study, bone marrow-derived MSCs used in rotator cuff repair enhanced healing rates and prevented reruptures over a 10-year period, showing substantial improvements in tendon integrity (Hernigou et al., 2014). In the treatment of patellar tendinopathy, bone marrow-derived stem cells showed significant improvements in tendon structure at 6 months compared to leukocyte-poor platelet-rich plasma (Rodas et al., 2021). In chronic tendinopathy, adipose-derived stem cells provided faster recovery in pain relief and functional improvement compared to SVF treatment (Lee et al., 2015). For non-insertional Achilles tendinopathy, both PRP and SVF treatments significantly improved clinical scores, but SVF-treated patients achieved faster improvements (Gomes et al., 2012). Stem cell therapy has demonstrated good safety and potential efficacy in tendon-related diseases. Although in some cases stem cell treatments did not show significant improvements over conventional treatments, they have shown considerable advantages in improving tendon structural integrity. We believe that in current clinical research, the means of using stem cell therapy is simply injection. No optimization methods such as improving the microenvironment or using induction factors have been considered. These are the factors that are studied more in basic medical research. Future research should continue to explore optimal strategies and long-term effects in clinical applications to fully leverage the regenerative potential of stem cells.

## 5 Conclusion and future outlook

In conclusion, the field of tendon tissue engineering, particularly involving TDSCs, is rapidly progressing, providing novel insights and strategies for addressing tendon and ligament injuries. The utilization of TDSCs in various therapeutic approaches, such as scaffold-based regeneration, has demonstrated promising results in enhancing tendon repair and function. Nonetheless, challenges remain in optimizing cell-scaffold interactions, sustaining long-term cell viability and function, and translating research findings into clinical applications. There are currently no clinical trials on TDSCs. Therefore clinical research is also very important in this field. Future research should concentrate on integrated approaches that combine advanced biomaterials, precise cellular manipulation, and a deeper understanding of the biomechanical environment of tendons. This multifaceted strategy possesses the potential to overcome the traditional challenges in tendon repair and regeneration, furnishing effective treatments for tendon injuries and disorders. As the discipline advances, it holds great promise for improving the outcomes of tendon repair, potentially revolutionizing the management of tendon and ligament injuries in clinical practice.
